# Process mapping of a collaborative quality improvement study to optimise the use of antibiotics in intensive care units in Argentina: a Pathfinder study

**DOI:** 10.12688/wellcomeopenres.26970.1

**Published:** 2026-08-13

**Authors:** Javier Roberti, Ana Paula Rodríguez, Viviana Rodríguez, Facundo Jorro-Barón, Ezequiel Garcia-Elorrio

**Affiliations:** 1 CIESP, National Scientific and Technical Research Council (CONICET), Buenos Aires, Autonomous City of Buenos Aires, Argentina; 2 Patient Safety and System Quality, Institute for Clinical Effectiveness and Health Policy, Buenos Aires, Autonomous City of Buenos Aires, Argentina

**Keywords:** Pathfinder, stewardships, critical care, implementation, capacity-building

## Abstract

**Objective:**

To systematically document the operational components, barriers, facilitators, and solutions of a quality improvement collaborative for antibiotic stewardship in intensive care units (ICUs) in the public sector of Argentina, using the Pathfinder methodological framework.

**Methods:**

Retrospective, descriptive observational study using mixed methods. Data sources included a documentary review of protocols, standard operating procedures, monitoring reports, and host study publications; an online survey with closed and open-ended questions administered to research team members; and triangulation with findings from the host study’s previously published process evaluation, structured according to Normalisation Process Theory. Integration was performed using the Pathfinder Tracker. Analysis combined descriptive statistics with content analysis of open-ended responses.

**Results:**

Eleven stages of the research process were mapped, from question formulation to dissemination, with an estimated total effort of approximately 1,020 person-hours. The stages with the highest perceived difficulty (4/5) were ethical and institutional approvals, data collection, analysis, and dissemination, which together accounted for approximately 50% of the total estimated effort. Barriers were identified across three domains: institutional and regulatory (heterogeneity of ethical requirements, absence of stable local coordinators, publication costs); operational and technical (complex data collection forms, displacement of implementation focus due to the burden of data collection, variable connectivity); and human resources and capacity (clinical workload, staff- -turnover, insufficient training in implementation science). Solutions included a collaborative format with mentoring, agile communication via accessible digital tools (REDCap, WhatsApp, Zoom), and the use of theoretical frameworks for structuring (NPT, CFIR).

**Conclusions:**

The Pathfinder framework enabled a systematic and transferable reconstruction of the host study’s processes, revealing barriers and solutions that conventional results reports do not capture. The findings are relevant for research teams in middle-income countries designing quality improvement collaboratives in complex hospital settings.

## Plain Language summary

A Pathfinder study is a new type of research specifically designed to document *how* a study was actually carried out. It maps each step of the research process, records how long each step took, and identifies difficulties and solutions. The aim is to create a guide that other research teams in similar settings can use.

This study reconstructed the processes behind the COST study. The COST study was a collaborative project designed to help teams of doctors, nurses and pharmacists in nine public-sector intensive care units across Argentina to improve how they prescribe antibiotics. We used three sources of information: a review of study documents, a survey of members of the research team, and previously published interviews with healthcare workers.

Eleven stages of the research process were mapped, from defining the research question through to publishing the findings, with an estimated total workload of approximately 1,000 person-hours. Difficulties identified: obtaining ethical and administrative approvals was burdensome. The data collection forms used in the study were overly complex, frustrating staff and leading to errors. The effort required to collect data distracted local teams from their main purpose: changing clinical practice. High clinical workloads, staff changes, and insufficient training in research methods limited teams' ability to participate fully. What worked: Virtual meetings and feedback kept centres engaged. Support from specialist societies gave the project credibility. Digital tools provided practical, low-cost solutions. Theoretical frameworks helped the team make sense of the results across different hospitals.

Research on improving healthcare in public hospitals in countries like Argentina faces recurring obstacles that rarely appear in published results papers. By documenting these obstacles and solutions, this Pathfinder study provides a practical resource for other research teams in Latin America and similar settings who are planning comparable projects.

## Introduction

Low- and middle-income countries carry a disproportionate burden of infectious diseases, yet contribute only a small share of the scientific evidence that shapes global health policy. ^1^ This gap reflects not only differences in infrastructure and funding, but also the absence of systematic processes that support clinical and epidemiological research in resource-limited settings. ^1^ ^,^ ^2^ Common barriers include weak research infrastructure, limited access to the literature, and the marginalisation of researchers from these countries as local collaborators rather than principal investigators. Research on implementing interventions to promote appropriate antibiotic use faces similar challenges, including shortages of human, financial, and technological resources, compounded by the absence of national antimicrobial-use guidelines. ^1^ ^–^ ^4^ Although local teams often develop practical ways to overcome these obstacles, such solutions are rarely documented. As a result, the gap persists: teams repeatedly encounter the same barriers without access to the lessons learned by others in the region. ^1^ ^,^ ^2^

The Pathfinder framework emerges as a response to this gap. ^5^ A Pathfinder is a complementary process mapping study that accompanies a ‘host study’—the main research—and documents its key stages, the challenges encountered, the solutions developed, and the tools used. Pathfinder studies can be prospective (documenting the process in real time) or retrospective (reconstructing the process after the host study concludes). Their central operational tool is a structured matrix that organises, by stage, the activities, time and resource metrics, barriers and solutions. ^5^ This study is the first Pathfinder specifically applied to collaborative quality-improvement research in intensive care units, focusing on the rational use of antibiotics in a Latin American country. It thus provides a quasi-experimental design context with an implementation process evaluation, which increases both the operational complexity and the relevance of this exercise.

The host study for this Pathfinder, known as COST, evaluated the implementation of an *antibiotic* stewardship programme in nine intensive care units (ICUs) within Argentina’s public healthcare sector, using a collaborative quality improvement process. The overall objective of this study was to reduce inappropriate antibiotic use in the ICU and increase antibiotic de-escalation. The design was an uncontrolled interrupted time series (16-week baseline, 32-week intervention) over 14 months. The intervention package included audits and feedback on antibiotic use, institutional clinical guidelines, antibiotic time-outs, interventions by the institutions’ pharmacy departments, and educational activities. The study was conducted in two learning sessions and three action periods, with mentoring and training aimed at improving quality. The results of the host study showed that implementation was feasible and improved key indicators, particularly de-escalation, with no statistically significant changes in days of therapy (DOT) or defined daily dose (DDD). ^6^ The study included a formative evaluation prior to the intervention and a process evaluation at the end of the intervention. ^7^

The guiding question of this Pathfinder is: Which critical processes of the host COST study needed to be understood and improved to strengthen future research on implementation and quality improvement in complex hospital settings? Its objective is to systematically document the operational components, barriers, facilitators and solutions implemented by the COST study research team, with a view to generating transferable knowledge for research teams in low- and middle-income countries.

## Methods

### General design

A retrospective, descriptive observational Pathfinder study was conducted using a mixed-methods design. ^5^The mapping was carried out based on the IECS in Buenos Aires, Argentina. The methodological framework of reference was that described by Uppal et al. ^5^

### Host study

The host study (COST) was a collaborative research project aimed at improving the quality of care and optimising antibiotic use in public ICUs in Argentina. The design was an uncontrolled interrupted time series across 9–10 ICUs over 14 months, with primary outcomes of DOT and DDD per 1,000 patient-days. The target population comprised ICU staff (doctors, pharmacists, nurses) and inpatients receiving antibacterial treatment. Full methodological details of the host study are described in the original publications. ^6^ ^,^ ^7^

### Data sources and procedures

The Pathfinder integrated three data sources using a convergent approach:

Document review. Protocols, timelines, standard operating procedures (SOPs) for data collection and uploading to REDCap, clinical audits, correspondence with ethics committees, technical monitoring reports, and publications derived from the host study were reviewed. The period covered all phases of the COST study, from problem formulation to dissemination of results.

Online survey. This was administered to members of the research team, including the central coordination unit (IECS), representatives of scientific societies (the Argentine Society of Intensive Care (SATI) and the Argentine Society of Infectious Diseases (SADI)), and site coordinators. The survey combined closed questions regarding the stages in which each person participated and open-ended questions, organised according to the stages of the Pathfinder Tracker, which gathered information on participation, difficulties encountered and their resolution, the resources and tools used, and lessons learnt at each stage.

The survey was conducted online in September 2025. Of the 11 members invited, all responded; 9 completed all sections, and 2 did not. The survey responses were cross-referenced with the findings of the COST study’s process evaluation, previously published, ^7^ which analysed 34 semi-structured interviews with healthcare workers from the participating ICUs using normalisation process theory (NPT). ^8^ ^,^ ^9^ As that evaluation was conducted on the same institutions, intervention and time period, its verbatim quotations are used in this Pathfinder as secondary material to triangulate and contextualise the survey findings and are explicitly identified as such. Within the framework of the Pathfinder, a small number of confirmatory interviews were also conducted; their content was consistent with the information already captured by the survey and did not yield any additional themes. The transcripts of these interviews were not available for analysis and therefore do not constitute a data source for this report.

Integration via the Pathfinder Tracker. The sources were consolidated into the Pathfinder Tracker matrix, organised by study phase. This integration enabled the identification of correspondences, complementarities and divergences between sources, with an emphasis on identifying barriers, facilitators and transferable solutions.

### Analysis

The quantitative component included descriptive statistics (absolute and relative frequencies, medians, ranges) applied to process metrics: estimated person-hours per stage and perceived level of difficulty according to the internal survey. The qualitative component consisted of a content analysis of the open-ended survey responses, organised deductively according to the stages of the Pathfinder Tracker and the categories of the survey itself (difficulties, resolutions, resources and lessons learnt). The responses were brief and explicit, so that the identification of barriers, facilitators and solutions required minimal interpretative inference; coding was used to consolidate and group the responses, not to construct theory. These findings were triangulated with the coded verbatim quotes from the process evaluation of the host study, previously published. ^7^ The integration was carried out in a convergent manner, organising the results by Tracker stages and regrouping them thematically by domain for presentation. Convergent triangulation across the internal survey, documentary sources and the host study's process evaluation served as the principal strategy for enhancing credibility; preliminary findings were discussed within the team.

This Pathfinder adopted a pragmatic, descriptive qualitative orientation, using deductive content analysis rather than an interpretivist theory-generating approach, consistent with the brief and concrete nature of the survey responses. As an insider study, it was designed and conducted by members of the COST research team. The survey was developed and administered by JR, PhD, a researcher at IECS with experience in implementation and qualitative research. This dual role afforded detailed knowledge of the processes mapped but constituted a potential source of interpretive bias, which was mitigated by triangulation against other sources and by structuring the analysis deductively against the Pathfinder Tracker. The internal survey was pilot-tested by two coordinators before administration. Open-ended responses were collated and coded by JR. Reporting follows the Standards for Reporting Qualitative Research (SRQR).

### Ethical considerations

The Pathfinder draws on data generated within the host study, which was conducted in accordance with the ethical and institutional approvals obtained from the Research Ethics Committees or Teaching and Research Committees of each participating hospital. The host study protocol explicitly provided that interviews would be conducted as part of the process evaluation; the qualitative material from that evaluation triangulated in this Pathfinder was generated under those approvals. The following approvals were granted: Hospital San Martín de La Plata (HSMLP2021/0048, 25 November 2021); Comisión Asesora en Investigación Biomédica en Seres Humanos de Neuquén (RIS34.00.21) and the Dirección de Bioética, Dirección Provincial de Organización de Establecimientos, Subsecretaría de Salud de Neuquén (Nota Nº 034/2022); Comité CIMEL (CBC-03-2022, 17 August 2022); Comité de Ética del Hospital de Infecciosas F. J. Muñiz (Nota 6015, 15 November 2021); CODEI, Hospital Pirovano (Nota 59, 10 November 2021); Comité de Ética del Hospital Rivadavia (Nota 6147, 10 January 2022); Comité de Ética del Hospital Simplemente Evita (Nota dated 7 April 2022); and the Comité de Ética en Investigación en Salud (CEIS), Ministry of Health of Jujuy Province (16 May 2022). The study was conducted in accordance with the principles of the Declaration of Helsinki, Resolution 1490/2007 of the Argentine Ministry of Health, and personal data protection regulations (National Law 25,326; Law 1845 of the Autonomous City of Buenos Aires). The primary data source for this Pathfinder was an internal survey of members of the research team concerning the operational conduct of the host study. This activity formed part of the methodological self-documentation of the research team's own work; it did not involve patients, third parties, or vulnerable groups, and it did not collect clinical, biological, or personal health data. On this basis, formal ethical approval or a waiver was not sought for the survey component, as it fell outside the scope of human-participants research requiring institutional review board oversight. Notwithstanding the survey's methodological nature, the study was conducted in accordance with the ethical principles of the Declaration of Helsinki and with national and city regulations on personal data protection (National Law 25,326; Law 1845 of the Autonomous City of Buenos Aires). Survey responses were attributed using role-based descriptors rather than personal identifiers to protect team members' confidentiality.

Participation in the survey was voluntary. All invited team members received written information describing the purpose of the survey, the operational (non-clinical) nature of the data sought, and the voluntary and confidential character of their participation, before accessing the questionnaire. Informed consent was obtained on an implied basis: voluntary completion and submission of the online questionnaire was taken to constitute consent to participate. Written signed consent was not sought because the survey posed no more than minimal risk, collected no sensitive personal or health data, and was administered remotely as a self-completed online instrument, for which an implied-consent procedure is proportionate and standard. No participants were minors.

## Results

### Participants

Of the 11 team members invited to take the survey, 9 completed all sections, including the open-ended questions. Among those who completed the survey, the distribution by role showed a predominance of central coordination: 4 from the IECS coordination team, 3 from scientific societies, and 2 hospital site coordinators. Those who completed the surveys were primarily involved in liaising with stakeholders, organising the work, and interpreting results, reporting and dissemination, whilst formative research and design selection were the least represented. This concentration of respondents in central coordination, compared with the limited representation of local ICU teams, should be borne in mind when interpreting the findings. The quotations presented below are taken from open-ended survey responses and the process evaluation of the host study, ^7^ used as secondary triangulation material.

### Operational components and process map

Eleven stages of the research and implementation process were mapped: (a) formulation of the research question, (b) choice of design, (c) obtaining ethical and institutional authorisations and approvals, (d) organisation of the work, (e) engagement with stakeholders and recruitment, (f ) formative evaluation, (g) data collection, (h) data analysis, (i) evaluation of the implementation process, (j) interpretation of results, and (k) report writing and dissemination. Table 1 summarises the operational components, key tools and estimated workload.

**Table 1.** Pathfinder Process Mapping Matrix.

Stage	Activities carried out (summary)	Time (person-hours)	Resources $ in addition to hours	Difficulties	Solutions	Tools	Perceived difficulty (1–5)	Residual risks
Question development	Literature review, initial meetings, definition of the problem and objectives.	24	May include access to databases	Time constraints, coordinating schedules.	Iterative meetings, use of shared drafts.	References, Word/Drive.	2	Lack of initial consensus.
Choice of design	Writing the protocol, defining indicators and the analysis plan.	90	NA	Time differences between authors.	Division of tasks, joint reviews.	Google Docs, Excel.	3	Underestimation of time required.
Approvals	Ethical, regulatory and institutional procedures.	112	Minimal (paperwork)	Varied requirements.	Standardised templates, active monitoring.	Consent forms, checklists.	4	Administrative delays.
Organisation and training	Formation of local teams, task schedule, training of collectors.	80	Low (connectivity and platform)	Workload overload, staff turnover.	Frequent virtual meetings, micro-tasks.	Zoom, WhatsApp, REDCap.	4	Reliance on local contacts.
Liaising with stakeholders	Recruitment of centres, institutional agreements and authorisations.	48	NA	Institutional heterogeneity.	Support from scientific societies, personalised follow-up.	SATI/SADI networks.	3	Loss of local commitment.
Formative evaluation	Design and conduct of 19 interviews, analysis, reports.	120	Low (transcription)	Difficulties in making contact, time constraints.	Flexible recruitment, training in implementation.	Atlas.ti, Zoom.	3	Heterogeneous participation.
Data collection	Uploading to REDCap, audits and central monitoring.	100	Hosting, servers (REDCap may be free)	Complex forms, upload delays.	Simplification of fields, continuous monitoring.	REDCap, WhatsApp.	4	Incomplete data.
Data analysis	Consolidation, time series models, quality review.	90	R institutional licence, Atlas.ti	Understanding the model, incomplete series.	Meetings with data unit, iterations.	R, Excel, Atlas.ti	4	Recording or adjustment errors.
Process evaluation	15 interviews, analysis, synthesis per centre.	120	Low (transcription)	Limited availability of local teams.	Flexible hours, multiple contacts.	Atlas.ti, CFIR matrices.	3	Self-selection bias.
Interpretation	Integration of quantitative and qualitative data, discussion with teams.	36	NA	Varying availability for meetings.	Mixed synchronous/asynchronous meetings.	Zoom, Google Drive.	2	Misalignment of results.
Dissemination	Drafting of reports, manuscripts and presentations.	200	Potential publishing costs	Time constraints, publication costs.	Report templates, editorial coordination.	Word, Paperpile, EndNote.	4	Delays in peer review.

The total workload for the study was approximately 1,000 person-hours, distributed among the central study coordination team—in this case, at the Institute for Clinical and Healthcare Effectiveness in Buenos Aires (≈45%)—the local teams in the ICUs (≈40%) and the analysis and dissemination of the results (≈15%). The qualitative components—formative evaluation and process evaluation (≈120 hours each)—together accounted for around one-fifth of the total workload and provided essential information for interpreting the mechanisms of implementation and adoption of the interventions. The most demanding stages were ethical and institutional approvals, data collection, analysis and dissemination, which accounted for over 70% of the work.

The study timeline spanned 23 months: the formulation of the research question and approvals began in months 0–1; the recruitment of centres and the organisation of local teams took place in months 2–3; training and piloting of instruments in months 4–5; the implementation of the collaborative in months 6–17; the analysis and integration of results in months 18–20; and the writing of reports and dissemination of results in months 21–23.

To ensure that this experience can be transferred to other teams facing similar conditions, we here list tools used during the study: the WHO Infection Prevention and Control Assessment Framework, which enables the assessment of institutional performance across eight domains prior to initiating an intervention of this type; the IHI Collaborative Assessment Scale, useful for monitoring the progress of participating teams; the Driver Diagram, which links the collaborative’s central objective with its primary and secondary drivers and improvement cycles; and the TIDieR. See extended data section.

Figure 1 shows the process of the COST host study, along with the perceived difficulty level of the participating researchers.

**Figure 1.**
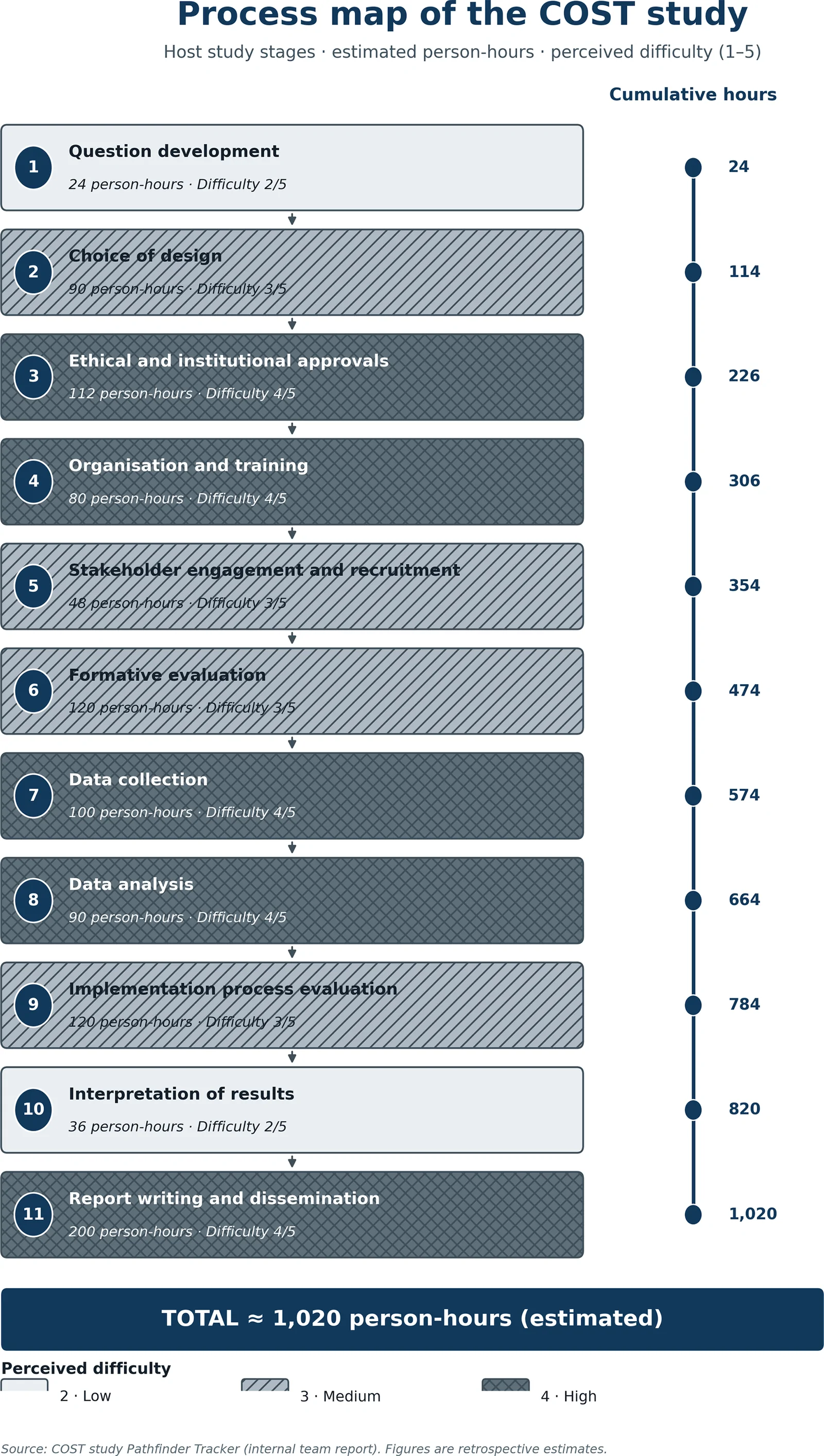
Map of the host study process.

### Barriers

The barriers identified were grouped into three thematic domains.

***Institutional and regulatory aspects***

The main barrier was the heterogeneity of ethical and institutional requirements across the 9–10 participating hospitals. Administrative processing times varied considerably between institutions; forms were not standardised, and individual follow-up was required at each site to ensure all approvals were obtained. This stage was rated as the most difficult (4/5), alongside data collection, analysis and dissemination. The difficulty at this stage was marked by a heavy administrative burden, a lack of timely and appropriate responses from the institutions required to authorise the study, the repeated submission of documentation, and the need for meticulous monitoring of the procedures initiated to obtain authorisations.

In the early stages of the study, the inclusion of scientific societies in the research group added regulatory and governance complexity, as noted by a member of the central coordination team:

*“The inclusion of other institutions in the research group, such as scientific societies, requires consideration of the time needed to analyse the legal aspects requested by each entity. It also requires clear frameworks of understanding that define roles, responsibilities, remuneration and authorship” (Central Coordination, IECS).*

From an institutional perspective, the absence of established local contacts in each of the participating hospitals constituted a cross-cutting barrier that had a cumulative impact: staff turnover in the ICUs affected the continuity of data recording, required repeated cycles of retraining and, in one case, led to a participating institution withdrawing from the study due to a lack of capacity to form a local team with sufficient institutional support. This difficulty arose throughout the study, not just during the initial team-building phase. The heterogeneity of the centres was highlighted by a representative of the scientific society:

*“The most significant difficulty was ensuring continuity of work with the centres. The institutions were heterogeneous, with varying resources and availability” (referring to SADI).*

Publication costs emerged as a persistent barrier during dissemination, with a direct impact on submission times and journal selection.

***Operational and technical***

The REDCap forms, which were initially complex, led to frustration among data collectors, data entry errors and delays in validation. Progressive simplification, field clarification, concrete examples, and the weekly audit protocol were associated with an estimated 30% reduction in data entry errors and shorter validation times. In this regard, a tension was observed between the logical requirements of the data analysis team conducting the audit and those of the data collection teams at the institutions. This interaction was mediated by the IECS research team. The complexity of the tools was repeatedly highlighted:

*“data collection form were very complex and that caused burnout among data collectors” (Data Analyst, IECS).*

The most significant conceptual barrier was the shift in the focus of implementation due to the burden of data collection. In several teams, the emphasis on data collection and validation—necessary to ensure the quality of the information—tended to divert attention away from the organisational change process itself. The teams perceived the intervention more as a recording exercise than as a process of improving clinical practice. This perception is consistent with the process evaluation of the host study, in which a healthcare worker described the initial difficulty in understanding the purpose of the intervention:

*“We are still maintaining all the changes implemented in the ward, from hand hygiene to the protocol for early removal of catheters and tubes” (UCI6, TS13)*

Linked to this finding, the results of the formative evaluation did not always lead to substantive adjustments to the intervention, highlighting a specific gap between knowledge generation and its application. The disconnect between knowledge generation and its application was clearly expressed by a researcher from the qualitative component:

*The people who weren't directly involved in the formative research often don't see the point of it. There's a sense that it gets done because it has to be done — and then the results just get filed away." (Qualitative researcher, IECS).*

Variable connectivity and technological access between sites caused delays in data uploading and limited participation in virtual meetings at certain institutions, particularly in the interior of the country.

***Human resources and capacities***

Clinical workload was the most frequent and pervasive barrier: it limited participation in learning sessions, timely data uploading and the availability of teams for interviews in both qualitative components. Staff turnover required repeated cycles of retraining that consumed time and resources at all stages of the study. The clinical workload and the difficulty in balancing tasks were described directly by a site coordinator:

*“Basically, the biggest difficulty is juggling multiple tasks, and academic or research work becomes difficult” (Site coordinator).*

In the process evaluation of the host study, this structural pressure was attributed to the country’s working context:

*“This is the reality in our country, where many healthcare workers have two or three jobs to make ends meet” (UCI2, TS1).*

Initial training in implementation science proved insufficient at several sites. This shortcoming hindered the local teams’ understanding of the purpose of formative and process evaluation, limiting their ability to actively participate in interpreting the results. Variations in prior experience with quality improvement across ICUs led to substantial differences in the time required to consolidate the role of change agent.

During the dissemination phase, delays in communication and feedback between team members affected the time taken to write and submit manuscripts. The concentration of multiple roles in a small number of coordinators created a risk of overload at various stages of the study.

### Facilitators and solutions

The collaborative, mentored format was the most important facilitating factor and had the greatest impact on the study. Fortnightly virtual learning sessions, PHEA (Plan–Do–Review–Act) cycles with educational follow-up, and regular feedback maintained adherence in 80% of participating centres. Co-design with a multidisciplinary approach—which involved intensive care specialists, infectious disease specialists, pharmacists and nurses—increased the relevance of the intervention and the commitment of local teams. The value of local support and regular feedback was highlighted across the board:

*“All continuous improvement projects require strong leadership from local teams; without that, it is impossible to make progress” (Facilitator, SATI)*

*“Recurring fortnightly meetings with continuous feedback […] scheduled feedback, with recurring meetings with a pre-determined agenda” (Central Coordination, IECS).*

Institutional networks served as enabling resources: SATI and SADI coordinated recruitment and lent legitimacy to the call for participation, whilst the IECS provided central coordination, technical capacity and data management. Letters of commitment signed by hospital and ICU authorities acted as a governance mechanism and as a prerequisite for sustained participation.

The accessible digital tools provided replicable solutions in similar contexts: REDCap for data management, WhatsApp and email to streamline communication, Zoom for virtual meetings, and Atlas.ti for qualitative analysis. Shared document editing on Google Drive, with explicit working rules, accelerated collaborative writing times during the dissemination phase.

The use of theoretical frameworks structured the analysis and interpretation of the qualitative results: the theory of the normalisation process guided the formative evaluation prior to the intervention, and the Consolidated Framework for Implementation Research (CFIR) guided the process evaluation, facilitating comparison between sites and the integration of findings. Both frameworks were coded in a complementary manner during the interpretation stage.

The transferable outputs generated by the study included the driver diagram, the prioritisation matrix, the PHEA cycles, the IPC Assessment Framework (WHO), semi-structured interview guides, data collection SOPs with operational definitions and examples, and a collaborative progress scale adapted from the IHI. These artefacts are available for reuse by teams undertaking similar studies. The sustainability of the changes was illustrated by a site coordinator:

*“We are still maintaining all the changes implemented in the ward, from hand hygiene to the protocol for early removal of catheters and tubes” (Site coordinator).*

These insights align with the process evaluation of the host study, in which the multi-centre collaborative format was valued as a source of emotional and technical support:

*“We are all trying to improve and we are not alone on this journey” (ICU4, TS11).*

## Discussion

This is the first Pathfinder study applied to collaborative research on quality improvement in the ICU. Systematic mapping enabled the documentation of processes that typically remain invisible in conventional monitoring systems: prior negotiations with hospital authorities, informal adjustments in response to staff rotations, or tensions between data production and the focus on changing practice. The total workload of approximately 1,000 person-hours provides a concrete benchmark for resource planning and can serve as a reference for teams planning similar studies.

Standard study monitoring systems, activity logs, progress reports, and data audits fail to capture certain processes—such as methodological adaptations—that lead to study outcomes. The Pathfinder methodology is specifically designed to make these processes visible. In the case of the COST study, the description of the steps, the tools used and the work carried out are not reflected in the main publications of the host study. Documenting this knowledge means that other teams facing similar conditions do not have to start from scratch and turns local experience into a transferable resource.

One of the most important findings is the shift in implementation focus due to the burden of data collection and, relatedly, the gap between the results of the formative evaluation and their translation into adjustments to the intervention. This phenomenon extends beyond the specific context of the COST study. In theoretical terms, the overload of recording tasks affected teams’ ability to deliver the intervention in real-world conditions and the shared coherence around its purpose. Furthermore, when the research coordination team fails to internalise the purpose and value of the knowledge generated by the formative evaluation—which had anticipated this difficulty—the feedback loop that should underpin the adaptation of the intervention is disrupted. It follows that the design of future projects of this type must establish protocols that translate formative findings into adaptation decisions and, furthermore, explicitly balance data production and practical implementation from the planning phase onwards.

Compared with the ZIPH-Track study, ^10^ which applied the Pathfinder framework to a prospective cohort of pregnant women in Honduras, structural barriers, staff turnover, regulatory heterogeneity and clinical overload are evident in both contexts, confirming their cross-cutting nature in countries with comparable circumstances. The differences are also worth noting: the COST study was conducted in an upper-middle-income country with a relatively robust technological infrastructure, but its complexity stems from the multimodal, multisite nature of the intervention and the tensions inherent in implementation studies embedded within collaborative research for improvement.

The findings of the COST study regarding staff turnover, the absence of protected time for research, and the lack of formal roles dedicated to research do not constitute isolated institutional problems, but rather expressions of a regional structural deficit. ^11^ These limitations are a direct consequence of a weak research infrastructure, the fragility of which becomes particularly visible in emergency situations: where locally rooted research capacity exists, it acts as a determining factor in enabling institutions to deploy timely diagnostic, genomic and epidemiological responses. Conversely, dependence on imported supplies and a shortage of trained personnel constitute structural vulnerabilities that compromise the sustainability of any intervention. ^11^ Documenting these barriers using the Pathfinder methodology is precisely the kind of foundational work needed to make future investments in research capacity in resource-limited settings transparent and justifiable.

The retrospective design implies a potential memory bias in the survey: processes are reconstructed from the present, and less prominent events may be underrepresented. The qualitative component of this Pathfinder was based on open-ended responses from the internal survey and on triangulation with the host study's process evaluation. Although the Pathfinder framework envisaged conducting a small number of confirmatory interviews, their content was consistent with the information already gathered in the survey and did not yield any additional themes. The Pathfinder did not include an analysis of direct costs; only items representing expenditure beyond working hours were included.

The actionable recommendations derived from this Pathfinder are: to simplify data collection forms from the design phase and pilot them before launch; to ensure stable local coordinators with clearly defined roles at each site; to formalise agreements on roles, timelines and authorship from the outset with all participating institutions and scientific societies; allocate explicit time and resources so that the findings of the formative evaluation are analysed by the team and translated into concrete adjustments to the intervention; and plan for publication funds from the budget formulation stage. The Pathfinder model applied to the COST study is replicable in other quality improvement collaborations in the region, and its adoption as a standard component can strengthen equity, institutional memory and the sustainability of implementation science in Latin America.

This Pathfinder study enabled a systematic and transferable reconstruction of the COST study processes, documenting operational components that conventional results reports do not capture and which are essential for other teams to plan similar studies. The estimated total workload provides a concrete reference point for resource allocation. The barriers identified, regulatory heterogeneity, clinical workload, and tension between data recording and improvement practice, are recognisable across multiple contexts of quality improvement in primary care and have documented, replicable solutions. The use of the Pathfinder as a standard component of collaborative research programmes can strengthen equity, institutional memory and the sustainability of implementation science in Latin America.

## Ethics and consent

The Pathfinder draws on data generated within the host study, which was conducted in accordance with the ethical and institutional approvals obtained from the Research Ethics Committees or Teaching and Research Committees of each participating hospital. The host study protocol explicitly provided that interviews would be conducted as part of the process evaluation; the qualitative material from that evaluation triangulated in this Pathfinder was generated under those approvals. The following approvals were granted: Hospital San Martín de La Plata (HSMLP2021/0048, 25 November 2021); Comisión Asesora en Investigación Biomédica en Seres Humanos de Neuquén (RIS34.00.21) and the Dirección de Bioética, Dirección Provincial de Organización de Establecimientos, Subsecretaría de Salud de Neuquén (Nota Nº 034/2022); Comité CIMEL (CBC-03-2022, 17 August 2022); Comité de Ética del Hospital de Infecciosas F. J. Muñiz (Nota 6015, 15 November 2021); CODEI, Hospital Pirovano (Nota 59, 10 November 2021); Comité de Ética del Hospital Rivadavia (Nota 6147, 10 January 2022); Comité de Ética del Hospital Simplemente Evita (Nota dated 7 April 2022); and the Comité de Ética en Investigación en Salud (CEIS), Ministry of Health of Jujuy Province (16 May 2022). The study was conducted in accordance with the principles of the Declaration of Helsinki, Resolution 1490/2007 of the Argentine Ministry of Health, and personal data protection regulations (National Law 25,326; Law 1845 of the Autonomous City of Buenos Aires). The primary data source for this Pathfinder was an internal survey of members of the research team concerning the operational conduct of the host study. This activity formed part of the methodological self-documentation of the research team's own work; it did not involve patients, third parties, or vulnerable groups, and it did not collect clinical, biological, or personal health data. On this basis, formal ethical approval or a waiver was not sought for the survey component, as it fell outside the scope of human-participants research requiring institutional review board oversight. Notwithstanding the survey's methodological nature, the study was conducted in accordance with the ethical principles of the Declaration of Helsinki and with national and city regulations on personal data protection (National Law 25,326; Law 1845 of the Autonomous City of Buenos Aires). Survey responses were attributed using role-based descriptors rather than personal identifiers to protect team members' confidentiality.

## Data Availability

Data associated with this study are not openly available due to the potential identifiability of individuals and the sensitive nature of the information related to operational roles, experiences, and perceptions within the COST study research team. Data access is restricted for ethical and confidentiality reasons, in accordance with the approved protocol. The research team remains committed to collaborating with other investigators interested in further analysis under controlled access conditions. Applications for access to the restricted data should be addressed to the corresponding authors and must include the purpose of the proposed analyses, the variables requested, data protection safeguards, and institutional affiliation. Requests will be reviewed by the study leadership team, and access may be granted upon approval and signature of a data use agreement, under conditions that ensure confidentiality and prevent re-identification of individuals. Repository: Zenodo. Published under a CC-BY 4.0 licence. DOI doi.org/10.5281/zenodo.20646044. ^12^ The following information is available under Zenodo. Published under a CC-BY 4.0 licence. DOI doi.org/10.5281/zenodo.20646044 ^12^: Survey instrument, invitation, blank Pathfinder tracker and SRQR checklist. Here are the practical instruments we used, please get them from the source: SQUIRE Guide (in Spanish) Standard for Quality Improvement Reporting Excellence https://www.equator-network.org/library/translations-of-reporting-guidelines/#Spanish Assessment Scale for Collaboratives by Institute for Healthcare Improvement https://www.ihi.org/library/tools/quality-improvement-essentials-toolkit Marco de Evaluación de prevención y control de infecciones al nivel de establecimientos de atención de salud de la Organización Mundial de la Salud. WHO Evaluation Framework in Spanish. https://www.paho.org/es/documentos/marco-evaluacion-prevencion-control-infecciones-al-nivel-establecimientos-atencion-salud Diagrama Conductal, Kit de Herramientas de Quality Improvement Essentials del Institute for Healthcare Improvement (Spanish) https://www.ihi.org/library/tools/quality-improvement-essentials-toolkit QI Essentials Toolkit Driver Diagram (English version), Institute for Healthcare Improvement. https://www.ihi.org/library/tools/quality-improvement-essentials-toolkit Template for intervention description and replication (TIDieR) checklist and guide. Tammy C Hoffmann T, Glasziou P, Boutron I,et al. Better reporting of interventions: template for intervention description and replication (TIDieR) checklist and guide. BMJ 2014;348:g1687 doi: 10.1136/bmj.g1687 https://www.bmj.com/content/348/bmj.g1687.long
