# Supplementary material for: Process mapping of a collaborative quality improvement study to optimise the use of antibiotics in intensive care units in Argentina: a Pathfinder study
Source: Wellcome Open Res. 2026 Oct 21;11:522. Originally published 2026 Aug 13. [Version 3] doi: 10.12688/wellcomeopenres.26970.3 (PMC13653102; doi:10.12688/wellcomeopenres.26970.3)
Supplement: Peer review history

# Reviewer report: Reviewer response for version 2

**DOI:** 10.21956/wellcomeopenres.30616.r170266

**Published:** 2026-10-09

**Authors:** Bode Ireti Shobayo

**Affiliations:** 1 Karolinska Institutet, Stockholm, Sweden

This manuscript is a retrospective Pathfinder study of the processes involved in the implementation of the COST antimicrobial stewardship quality improvement collaborative across intensive care units in Argentina and systematically documents the operational processes. The authors used a mixed-methods approach combining a document review, an internal survey of research team members, and triangulation with a previously published process evaluation in an effort to map eleven stages of implementation, estimate workload requirements, identify barriers and facilitators, and generate lessons for future implementation science projects in low- and middle-income countries. The study estimates a workload of approximately 1020 person-hours and identifies regulatory challenges, data collection burden, staff turnover, limited implementation science capacity and competing clinical priorities as significant barriers. The authors conclude that Pathfinder studies can develop transferable operational knowledge that is typically absent from traditional effectiveness studies and can bolster implementation research capacity in resource-limited settings.

 It would not affect the scientific validity to reduce the repetition of terms such as “transferable knowledge”, “transferable solutions” and “transferable resource” to improve readability. The discussion is a little repetitive regarding staffing shortages and barriers to implementation, and could be more concise.

 Although the survey instrument is available as supplementary data, the manuscript itself provides limited information concerning total number of survey questions, distribution of closed versus open-ended questions, and how pilot-testing influenced the final instrument. Future researchers attempting to replicate the study may find these details useful.

 The manuscript states that findings from three data sources were triangulated, but the actual integration process remains somewhat unclear.

 Specifically, how were discrepancies between sources resolved? What criteria were used to determine convergence? Were contradictory findings encountered?

**Approve with Minor Revisions**

Is the work clearly and accurately presented and does it cite the current literature?

Yes

If applicable, is the statistical analysis and its interpretation appropriate?

Yes

Are all the source data underlying the results available to ensure full reproducibility?

Partly

Is the study design appropriate and is the work technically sound?

Yes

Are the conclusions drawn adequately supported by the results?

Yes

Are sufficient details of methods and analysis provided to allow replication by others?

Partly

Reviewer Expertise:

Antibiotic Resistance, Infectious Disease Epidemiology

I confirm that I have read this submission and believe that I have an appropriate level of expertise to confirm that it is of an acceptable scientific standard.

# Response

**Published:** 2026-10-14

**Authors:** Javier Roberti

**Affiliations:** Patient Safety, Institute for Clinical Effectiveness and Health Policy, Buenos Aires, Autonomous City of Buenos Aires, Argentina

We thank the reviewer for a careful reading and for the constructive suggestions. We have revised the manuscript (version 3) as follows.

***Comment 1: It would not affect the scientific validity to reduce the repetition of terms such as “transferable knowledge”, “transferable solutions” and “transferable resource” to improve readability. The discussion is a little repetitive regarding staffing shortages and barriers to implementation and could be more concise.***

**Response:** We thank the reviewer for this suggestion. We have reduced the use of “transferable” throughout the manuscript and retained it only where it defines the study objective (Introduction). We have also shortened the Discussion. The paragraph on staffing shortages and research-capacity deficits has been condensed so that it no longer repeats barriers already reported in the Results. We have removed a sentence on equity, institutional memory and sustainability that appeared twice, once at the end of the recommendations and again in the closing paragraph. We have also removed from the closing paragraph a sentence restating the barriers.

***Comment 2: Although the survey instrument is available as supplementary data, the manuscript itself provides limited information concerning total number of survey questions, distribution of closed versus open-ended questions, and how pilot-testing influenced the final instrument. Future researchers attempting to replicate the study may find these details useful.***

**Response: **We thank the reviewer for this suggestion and have added a full description of the instrument to the Methods. The questionnaire comprised six items. The first was a closed, multiple-choice item recording the stages of the host study in which the respondent had participated. The other five were open-ended items asking respondents to describe, for each stage, their participation, the difficulties encountered, how these were resolved, the resources used and the lessons learnt most useful to other teams. Respondents were asked to write a separate paragraph for each stage. The predominantly open format was deliberate. The Pathfinder was originally intended to conduct in-depth interviews with all team members. When this proved unfeasible, the questionnaire was designed to reproduce the prompts of an interview guide in self-completed form. Alongside the questionnaire, respondents rated the difficulty of each stage in which they had participated on a five-point Likert scale. We also clarify that stage-level person-hour estimates were obtained in interviews with team researchers and the project supervisor.

**Comment 3: The manuscript states that findings from three data sources were triangulated, but the actual integration process remains somewhat unclear. Specifically, how were discrepancies between sources resolved? What criteria were used to determine convergence? Were contradictory findings encountered?**

**Response: **We agree that the integration process needed to be made explicit and have rewritten the corresponding section of the Methods. The three sources were entered stage by stage into the Pathfinder Tracker and each finding was classified as convergent, complementary or dissonant: **Convergent:** the same barrier, facilitator or solution was reported in at least two sources. **Complementary:** a finding was reported in one source only, was not contradicted by another, and added detail. **Dissonant:** sources gave incompatible accounts. Documentary records were treated as the reference. The survey and the process evaluation were treated as the sources for experiential accounts. Discrepancies were to be resolved by returning to the documentary record and, where they could not be resolved, both accounts would be reported. No direct contradictions were identified. The principal difference between sources was one of emphasis: the internal survey foregrounded regulatory and administrative barriers, whereas the front-line interviews of the process evaluation foregrounded clinical workload. We treated this as complementarity, consistent with the limitation already reported on respondent composition. These results are now reported at the beginning of the Barriers section

# Reviewer report: Reviewer response for version 2

**DOI:** 10.21956/wellcomeopenres.30616.r169733

**Published:** 2026-09-24

**Authors:** Sarunyou Chusri

**Affiliations:** 1 Prince of Songkla University, Songkla, Thailand

I have reviewed the revised manuscript (Version 2) alongside the authors' comprehensive point-by-point responses to my previous comments.

 The authors have successfully addressed all of my concerns, particularly by clarifying the over-representation of the central coordination team to properly contextualize potential directional bias and by consistently reconciling the person-hour metrics to 1,020 hours as an explicit retrospective estimate.

 Furthermore, the discussion has been significantly strengthened by bridging process management with clinical realities—specifically linking operational friction to the emergence of resistant pathogens like *Acinetobacter baumannii*—and by extending the relevance of the Pathfinder framework to broader healthcare delivery models.

 With all formatting, grayscale legibility, and terminology issues thoroughly resolved, the manuscript is now highly polished, technically sound, and offers valuable, transferable insights for implementation science in resource-constrained settings. I am completely satisfied with this revision and enthusiastically endorse this work for indexing.

Is the work clearly and accurately presented and does it cite the current literature?

Partly

If applicable, is the statistical analysis and its interpretation appropriate?

Partly

Are all the source data underlying the results available to ensure full reproducibility?

Partly

Is the study design appropriate and is the work technically sound?

Partly

Are the conclusions drawn adequately supported by the results?

Partly

Are sufficient details of methods and analysis provided to allow replication by others?

Partly

Reviewer Expertise:

Infectious Disease ; Antimicrobial Resistance

I confirm that I have read this submission and believe that I have an appropriate level of expertise to confirm that it is of an acceptable scientific standard.

# Reviewer report: Reviewer response for version 1

**DOI:** 10.21956/wellcomeopenres.29691.r167156

**Published:** 2026-09-10

**Authors:** Sarunyou Chusri

**Affiliations:** 1 Prince of Songkla University, Songkla, Thailand

**General Comments**

 This manuscript presents a highly innovative application of the Pathfinder methodological framework to evaluate the implementation processes of a collaborative quality improvement project (the COST study) aimed at optimizing antibiotic stewardship within public sector intensive care units (ICUs) in Argentina. While conventional clinical trials or process evaluations capture pre-and-post indicators, they frequently omit the operational friction, administrative hurdles, and workload metrics required to execute complex healthcare interventions in middle-income countries. By utilizing a descriptive, mixed-methods approach integrated via the Pathfinder Tracker, the authors successfully quantify the human effort (~1,020 person-hours) and map distinct operational bottlenecks across 11 key research stages. The manuscript is well-structured, written with clarity, and provides highly practical, transferable insights for global health implementation science.

**Major Comments**

- Over 44% of the survey respondents were from the central coordination team (IECS), while hospital site coordinators represented a minimal fraction of the feedback. This skews the perceived difficulty scores and barrier identification toward administrative and operational logistics rather than clinical bedside realities.
- The calculation of exactly 1,020 person-hours requires a clearer breakdown. The authors should explicitly state how these retrospective estimates were validated against actual time logs or if they rely purely on post-hoc subjective recall via the survey.
- To enhance the impact and generalizability of this operational blueprint, the discussion must explicitly bridge the gap between process management and the global clinical consequences of stewardship failure.

1. The authors note that the primary clinical focus of the host COST study was reducing inappropriate antibiotic use and increasing de-escalation. The gravity of failing to execute these operational steps is underscored by Chusri et al. (2015), who demonstrated that long-term broad-spectrum exposures and even short-term carbapenem use directly multiply the hazards of acquiring highly lethal nosocomial carbapenem-resistant *Acinetobacter baumannii* (CRAB) infections. Incorporating this clinical baseline underscores *why* reducing operational friction in data collection is globally vital: delays in program implementation directly correspond to a rise in untreatable ICU pathogens.
2. Furthermore, while this Pathfinder study maps a complex multimodal ICU intervention, the operational barriers identified—such as insufficient training and structural work overload—echo systemic vulnerabilities seen at all levels of healthcare globally. This pattern is validated by Chang et al. (2019), who characterized rampant patterns of unnecessary prescribing, extended-spectrum escalation, and unindicated combined antibiotic usage in resource-constrained primary healthcare hospitals.

By contrasting the intensive infrastructure of Argentine public ICUs with the primary care clinical patterns identified by Chang et al. (2019), the manuscript can argue that the Pathfinder framework is not merely a niche tool for specialized trials, but an essential tool required to diagnose infrastructure deficits and optimize stewardship across vastly diverse global health delivery models.

 Reference

1. Refer (1)
2. Refer (2)

**Minor Comments**

- On page 2, column 1, paragraph 2, remove the double hyphen artifact in the phrase " **staff- -turnover**".
- Ensure that the color-coding hierarchy for difficulty thresholds (Diff. 2 Low vs Diff. 4 High) features sufficient contrast for grayscale print readability.
- The methods section mentions both "implied consent" for online submissions and "implied basis". Standardize the terminology to clearly reflect standard institutional review board (IRB) nomenclature.

Is the work clearly and accurately presented and does it cite the current literature?

Partly

If applicable, is the statistical analysis and its interpretation appropriate?

Partly

Are all the source data underlying the results available to ensure full reproducibility?

Partly

Is the study design appropriate and is the work technically sound?

Partly

Are the conclusions drawn adequately supported by the results?

Partly

Are sufficient details of methods and analysis provided to allow replication by others?

Partly

Reviewer Expertise:

Infectious Disease ; Antimicrobial Resistance

I confirm that I have read this submission and believe that I have an appropriate level of expertise to confirm that it is of an acceptable scientific standard, however I have significant reservations, as outlined above.

## References

[1] Chusri S, Silpapojakul K, McNeil E, Singkhamanan K, Chongsuvivatwong V. Impact of antibiotic exposure on occurrence of nosocomial carbapenem-resistant Acinetobacter baumannii infection: a case control study. J Infect Chemother. 2015 Feb;21(2):90-5. 10.1016/j.jiac.2014.10.00225454216

[2] Chang Y, Chusri S, Sangthong R, McNeil E, Hu J, Du W, Li D, Fan X, Zhou H, Chongsuvivatwong V, Tang L. Clinical pattern of antibiotic overuse and misuse in primary healthcare hospitals in the southwest of China. PLoS One. 2019 Jun 26;14(6):e0214779.10.1371/journal.pone.0214779PMC659457631242185

# Response

**Published:** 2026-09-14

**Authors:** Javier Roberti

**Affiliations:** Patient Safety, Institute for Clinical Effectiveness and Health Policy, Buenos Aires, Autonomous City of Buenos Aires, Argentina

We thank the reviewer for a constructive appraisal of our manuscript. We have addressed each comment below. Reviewer comments are given in italics; our responses follow, with the location of each change in the revised manuscript (all changes are shown as tracked changes).

***Comment 1. Over-representation of the central coordination team (IECS) among survey respondents, which may skew perceived-difficulty scores and barrier identification towards administrative and operational logistics rather than clinical bedside realities.***

**Response:** We agree, and we consider this the most important limitation to make explicit for the reader. We have not altered the data, but we have sharpened the way the skew is reported in two places and specified the likely *direction* of the resulting bias. In the Results (Participants), we now state that the concentration of respondents in central coordination (four of nine completers) relative to local ICU site coordinators (two of nine) is an interpretive limitation that shapes the barrier profile, and that the perceived-difficulty ratings and identified barriers are weighted towards administrative, regulatory and operational realities and are likely to under-represent bedside and ward-level barriers experienced by front-line clinical staff. In the Discussion (Limitations), we have added a dedicated statement to the same effect, noting that triangulation with the host study’s independently conducted process evaluation, based on 34 interviews with front-line healthcare workers, was used to partially counterbalance this skew, while making clear that the internal survey findings should not be read as a balanced representation of all participant perspectives.

***Comment 2. The calculation of exactly 1,020 person-hours requires a clearer breakdown, and the authors should state whether these retrospective estimates were validated against actual time logs or rely on post-hoc subjective recall.***

**Response: **We thank the reviewer. This helped us correct inconsistencies in the previous version. We have made three changes. First, we now state explicitly and consistently throughout (Abstract, Results, Discussion and Plain Language Summary) that the total is approximately 1,020 person-hours, corresponding exactly to the sum of the eleven stage-level estimates in Table 1. The previous discrepancy between “≈1,000” in the text and 1,020 in the table has been removed. Second, and directly in response to the reviewer’s question, we now state that these figures are retrospective estimates provided by respondents through the internal survey; that no independent activity-tracking records were maintained during the host study; and that the estimates were therefore not validated against measurement and should be read as an order-of-magnitude planning benchmark. Third, we have replaced the previous role-based apportionment (which could not be reconstructed from the stage-level data in Table 1) with a stage-based breakdown that is fully traceable to the table: the four stages rated most difficult (ethical and institutional approvals (112 person-hours), data collection (100), data analysis (90) and dissemination (200))  together account for about half of the estimated effort (502 person-hours; 49%), and the two qualitative components (formative and process evaluation, about 120 person-hours each) account for around one-quarter of the total. Figure 1 has also been revised so that the reported total (1,020 person-hours) is consistent with the text.

**Comment 3. The Discussion should bridge process management and the global clinical consequences of stewardship failure, incorporating the clinical baseline (Chusri et al., 2015) to underscore why reducing operational friction is clinically vital.**

**Response: **We agree that the clinical stakes of stewardship should be made explicit, and we have added a passage to the Discussion establishing this link. We note that the host study’s clinical rationale bears on the emergence of resistant nosocomial pathogens, and we cite Chusri et al. (2015) to support the association between prolonged broad-spectrum and carbapenem exposure and the risk of acquiring carbapenem-resistant *Acinetobacter baumannii* infection in the ICU. We have framed this deliberately as *background motivation* for attending to process barriers, and we state explicitly that it is not an inference from the present study, which was not designed to link operational metrics to clinical endpoints. We have taken this framing to avoid overstating a causal chain.

**Comment 4. The manuscript should argue that the Pathfinder framework is not a niche tool but is relevant across diverse health delivery models, contrasting the Argentine ICU setting with primary-care prescribing patterns (Chang et al., 2019).**

**Response:** We have added a statement to the Discussion noting that several of the operational barriers identified here are not confined to the ICU or to research settings and citing Chang et al. (2019) as an illustration of comparable patterns of antibiotic overuse and misuse under conditions of limited training and constrained resources. We suggest that the process-mapping approach may be useful beyond specialised trials in diagnosing infrastructural and workforce deficits across levels of care. We hope the reviewer agrees that this measured formulation is preferable to a stronger claim of universality.

 Minor comments

**Comment 1. Remove the double-hyphen artifact in “staff- -turnover”.**

**Response: **Corrected to “staff turnover” (Abstract).

**Comment 2. Ensure the difficulty colour-coding has sufficient contrast for grayscale print readability.**

**Response: **Figure 1 has been redrawn so that the three difficulty tiers are distinguished redundantly by (i) fill shade, (ii) hatch pattern (none / diagonal / cross-hatch) and (iii) an explicit numeric difficulty label within each stage box. The encoding is therefore fully legible in greyscale and does not rely on colour alone. The legend has been updated accordingly.

**Comment 3. Standardise the consent terminology to conventional IRB nomenclature.** **Response:** We have standardised the wording in the Ethics section to describe the procedure as implied (opt-in) consent, obtained through voluntary completion and submission of the online questionnaire. The terminology is now internally consistent and aligned with the survey instrument provided as extended data During revision we identified and corrected a broken reference placeholder in the previous version (an “invalid citation”) and ensured that all in-text citations resolve correctly.

 The two references requested by the reviewer (Chusri et al., 2015; Chang et al., 2019) have been added to the reference list in citation order.

 We are grateful for the reviewer’s time and believe the manuscript is substantially strengthened by these revisions.

  Regards, Javier Roberti
